# Comparison of Medium Manganese Steel and Q345 Steel on Corrosion Behavior in a 3.5 wt % NaCl Solution

**DOI:** 10.3390/ma10080938

**Published:** 2017-08-11

**Authors:** Guanqiao Su, Xiuhua Gao

**Affiliations:** The State Key Laboratory of Rolling and Automation, Northeastern University, Shenyang 110819, China; suhang_joe@163.com

**Keywords:** corrosion rate, medium-Mn steel, SEM, EPMA

## Abstract

A cyclic wet/dry accelerated corrosion test was used to compare the corrosion behavior of medium-Mn steel and Q345 steel. In terms of scanning electron microscope (SEM), using X-ray diffraction (XRD), electron probe microanalysis (EPMA), X-ray photoelectron spectroscopy (XPS), and analysis of the corrosion process, the results showed that the medium-Mn steel did not exhibit higher corrosion resistance than Q345 steel due to the greater content of Mn-rich compounds in the rust layer. Moreover, the effect of a small amount of anti-corrosion elements in medium-manganese steel can regulate the corrosion rate. The conceptual model of the corrosion process of the medium-Mn steel in a 3.5 wt % NaCl solution is proposed.

## 1. Introduction

As the world continues development in the exploration and exploitation of offshore oil and gas resources, a large number of high-quality steel materials will replace the traditional offshore platform steel. Due to its low cost and the high strength and toughness, low-carbon medium-manganese steel will play a significant role in the construction of marine engineering projects [1,2,3]. The corrosion resistance is usually improved with the increase of anti-corrosion element contents. Nickel was added to the steel to achieve a synergistically-enhancing effect, with small amounts of copper enriching the outer layer of the steel substrate [4,5]. Molybdenum may generate insoluble FeMoO_4_ and become enriched near the outer layer of the corrosion products to serve as a barrier against corrosive ions during the corrosion process [6,7]. The presence of chromium in steels can decrease the corrosion rate through the formation of a compact barrier in the rust film with the formation of a Cr-rich compound. However, this steel may be adversely affected by the seawater environment owing to the lower content of the above anti-corrosion elements. Therefore, it becomes very important to the assessment of the corrosion behavior of this type medium-manganese steel.

In the new type medium-Mn steel, alloying with 5% Mn plays a significant role in enhancing the homogeneity of the mechanical properties, and it also improves the hardenability during the heat treatment process [8]. However, in terms of corrosion, few reports showed the effects of Mn on this steel. In consideration of the importance of Mn as an alloying element in new medium-manganese steel, the corrosion behavior of medium-manganese steel under a simulated seawater environment (a 3.5 wt % NaCl solution) was elaborated, and the Q345 steel was introduced into the thesis as reference materials.

## 2. Experimental Procedure

### 2.1. Preparation of the Tested Steels

The chemical compositions of medium-Mn steel and Q345 steel are listed in Table 1. In the manufacturing procedures, the medium-Mn steel was melted using a vacuum induction furnace (Benxi Steel Group Corporation, Benxi, China), and was then casted into ingots. In the following procedure, they were forged into billet slabs with a 140 mm thickness. The slab was heated to 1200 °C for 2 h and then hot rolled into a plate with a thickness of 30 mm under multiple passes from 960 to 940 °C. Subsequently, the plate was directly water-quenched to room temperature using an accelerated cooling system. Next, the plate was intercritically annealed for 60 min at 650 °C. Then the plate was air-cooled at 25 °C. The yield strength, ultimate tensile strength, and impact absorbed energy at −60 °C of the tested medium-Mn steel are 737 MPa, 868 MPa, and 206 J, respectively. The Q345 ingot was also prepared by the vacuum induction furnace in the laboratory. The yield strength of Q345 steel is ~345 MPa.

### 2.2. Cyclic Wet/Dry Accelerated Corrosion Test

The coupons cut from the two tested steels (60 mm × 40 mm × 4 mm and 20 mm × 15 mm × 4 mm) were subsequently polished with 240, 400, 600, and 800 grit silicon carbide papers, respectively. Those two types of coupons were then cleaned with petroleum ether, alcohol, and acetone using an ultrasonic cleaner and dried with cold air. Then the coupons (60 mm × 40 mm × 4 mm) were weighed using a balance with a precision of 0.1 mg and stored in a desiccator before the cyclic wet/dry experiment. A 3-mm diameter hole was machined to hang the coupons with a non-metallic wire.

According to EN ISO 11130 [9], the tested solution with a volume of 90 L contained 3.5 wt % NaCl, which was made from deionized water and analytical reagents. The pH value of the solution was around 6.5. Each wet/dry cycle consisted of a drying period (50 min at 25 °C) plus a wetting period (10 min at 25 °C). NaOH was provided to maintain the simulated seawater corrosive solution’s pH value. The accelerated corrosion test was divided into six parts, 24, 72, 168, 288, 432, and 600 h, respectively. Four replicate coupons were used for each corrosion test. Another important factor to consider was the humidity which, in the airtight container, should be maintained at 45% to create a humid environment. After the corrosion tests, the larger coupons (60 mm × 40 mm × 4 mm) were removed from the corrosion products to determine the mass loss. The coupons were pickled in 20% hydrochloric acid (HCl), inhibited with 20 g/L of hexamethylenetetramine (urotropine) and 500 mL distilled water, rinsed subsequently in deionized water and acetone, and dried [10]. Then, the mass loss of those coupons was used to calculate the corrosion rate.

### 2.3. Morphology Observation and Composition Analysis

The corrosion products of the two tested steels were observed by a FEI QUANTA 600 scanning electron microscope (SEM) (Hillsboro, OR, USA). Corrosion phases were detected by using a D/max-2400 X-ray diffraction (XRD) (Rigaku, Tokyo, Japan) with Cu Kα radiation and a step of 0.04°, and identified by matching peak positions automatically with MDI Jade software (Livermore, CA, USA) equipped with PDF-2 (2004). A JEOL-8530F electron probe analyzer (EPMA) (Tokyo, Japan) was set to a voltage of 20 kV and an 11 mm working distance to observe the elemental distribution. The composition of the corrosion films was detected with a Thermo ESCALAB 250 X-ray photoelectron spectroscopy (XPS) system (Waltham, MA, USA).

## 3. Results and Discussion

### 3.1. Corrosion Rate

In general, corrosion rate reflects the corrosion process of the materials. In this experiment, the corrosion rates were calculated according to the weight loss (Δ*W*, g) based on the following equation [11]

(1) Corrosion rate (mm/y) = (K × ΔW) / (A × T × D)

where *K* = 87,600, a constant for the corrosion rate evaluated in mm/year, *A* is the total corrosive area in cm^2^, *T* is the exposure time in hours, and *D* is the density of the tested steels in g/cm^3^.

**Figure 1:**
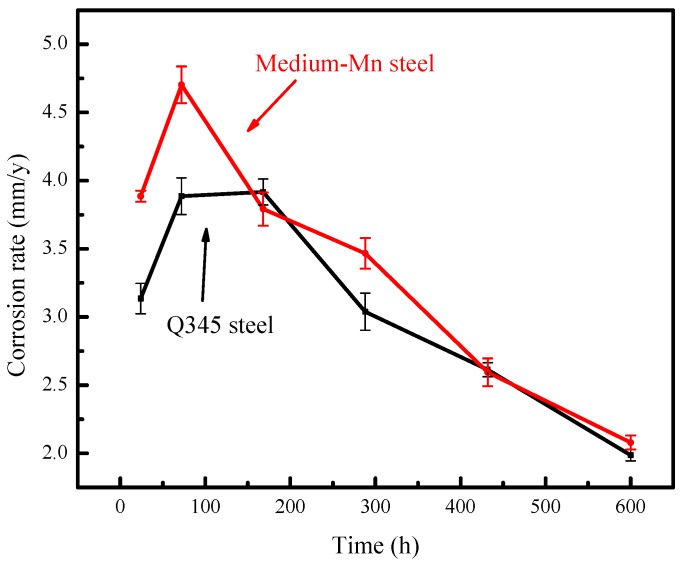
Corrosion rates of the tested medium-Mn steel and Q345 steel exposed to a 3.5 wt % NaCl solution for different lengths of time at 25 °C.

The corrosion kinetic curves for the two types of steel after cyclic wet/dry accelerated corrosion tests with a 3.5 wt % NaCl solution are presented in Figure 1. For the two kinds of tested steels, the overall trend of the corrosion rates versus corrosion time was similar. After 24 h and 72 h of corrosion time, the corrosion rate of the tested medium-Mn steel was higher than that of the Q345 steel and the differences were close to 1 mm/year. The corrosion rates of the two tested steels from 168 to 600 h exhibited slight changes. The final corrosion rate values of the tested medium-Mn steel and the Q345 steel in simulated seawater are 2.07 mm·year^−1^ and 1.99 mm·year^−1^, respectively.

### 3.2. X-ray Diffraction

**Figure 2:**
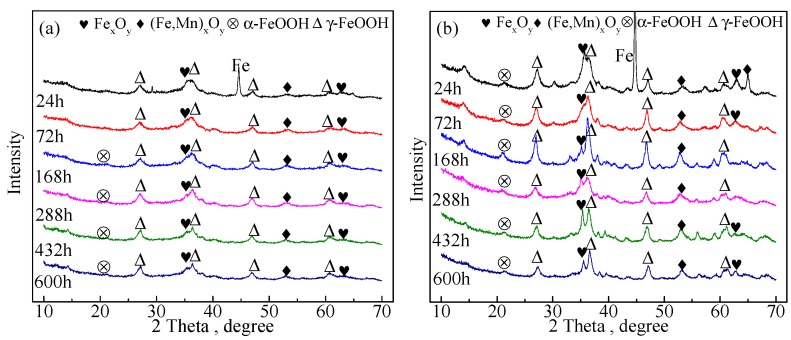
XRD patterns of the tested coupons after different corrosion times exposed to a 3.5 wt % NaCl solution for different lengths of time at 25 °C: (**a**) medium-Mn steel; and (**b**) Q345 steel.

Figure 2 presents the XRD patterns of the corrosive products taken from the surface of the two tested steels after corrosion tests of various durations. The corrosion phases of the medium-Mn steel and the Q345 steel are presented in Figure 2a,b, respectively. After the 24 h corrosion test, the steel substrate peak was larger than that of the other corrosive phase and that in Figure 2a was lower than that in Figure 2b. Other peaks mainly indicated the lepidocrocite (γ-FeOOH), small amounts of (Fe,Mn)_x_O_y_, Fe_x_O_y_, and goethite (α-FeOOH). The critical peaks of lepidocrocite and goethite corrosion phases increased after 168 h of corrosion time, respectively. However, in the final three corrosion durations, these peaks started to shrink. Additionally, the critical peaks of (Fe,Mn)_x_O_y_ and Fe_x_O_y_ did not change detectably over the entire corrosion process.

### 3.3. SEM Morphology

**Figure 3:**
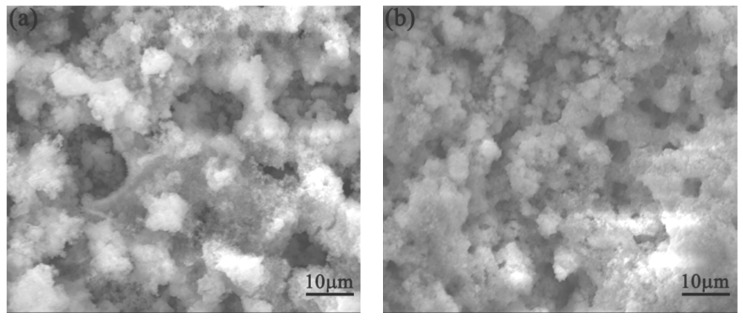

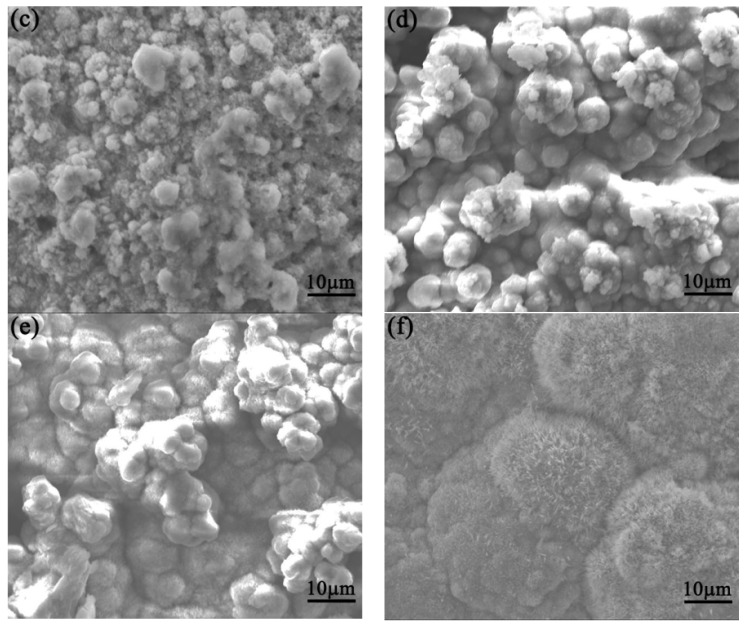
SEM morphologies of the tested medium manganese steel after different immersion durations: (**a**) 24 h; (**b**) 72 h; (**c**) 168h; (**d**) 288 h; (**e**) 432 h; and (**f**) 600 h.

**Figure 4:**
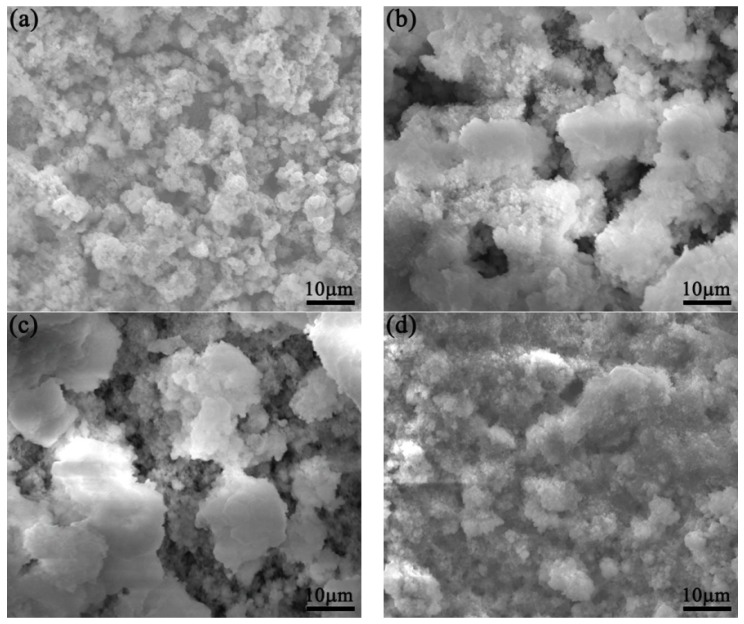

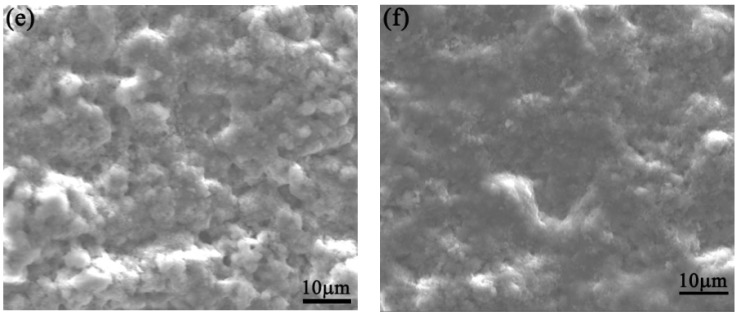
SEM morphologies of the tested Q345 steel after different immersion durations: (**a**) 24 h; (**b**) 72 h; (**c**) 168h; (**d**) 288 h; (**e**) 432 h; and (**f**) 600 h.

Figure 3 and Figure 4 show SEM images of surface morphologies of the tested medium-Mn steel and Q345 steel after cyclic wet/dry accelerated corrosion testing with a 3.5 wt % NaCl solution. After 24 h corrosion (Figure 3a and Figure 4a), the rust presented some flaky structures. The flaky structure was typical of γ-FeOOH [12]. Due to the existence of γ-FeOOH, the rust became loose and porous. The rust of the Q345 steel was denser than that of the tested medium-Mn steel. Therefore, the corrosion rate of tested medium-Mn steel was higher at the initial time. At the second time point, the γ-FeOOH mainly occupied the surfaces of the two tested steels. The size of γ-FeOOH above the medium-Mn steel was smaller than that of the Q345 steel (see Figure 3b and Figure 4b). Thus, this phenomenon may cause the gap in the corrosion rates of the two tested steels. As seen in Figure 3c and Figure 4c, the rust above the medium-Mn steel was denser than that of the previous one with the increasing corrosion time. It was also denser than the rust of the Q345 steel. The change at the morphology of the rust above the medium-Mn leads to the corrosion rate decreasing sharply. Furthermore, the rust of the Q345 was similar to that in the previous time point. Thus, the corrosion rate of the Q345 steel remained stable from 72 h corrosion to 168 h corrosion. As shown in Figure 3d,e and Figure 4d,e, the rust morphologies presented as a ‘whisker’ morphology, which was a typical of α-FeOOH [13]. The transformation of FeOOH could decrease the corrosion rate. Finally, the rust morphologies above the two tested steels achieved a smooth and dense state (see Figure 3f and Figure 4f), and the corrosion rate of the two tested steels achieved stable values.

### 3.4. EPMA Results

**Figure 5:**
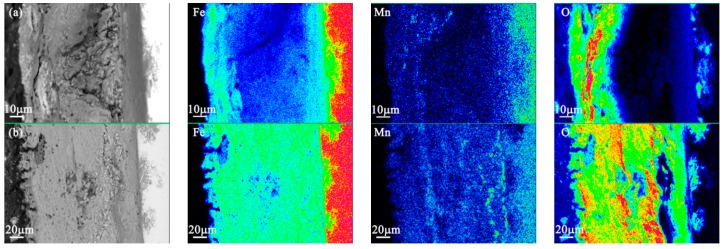

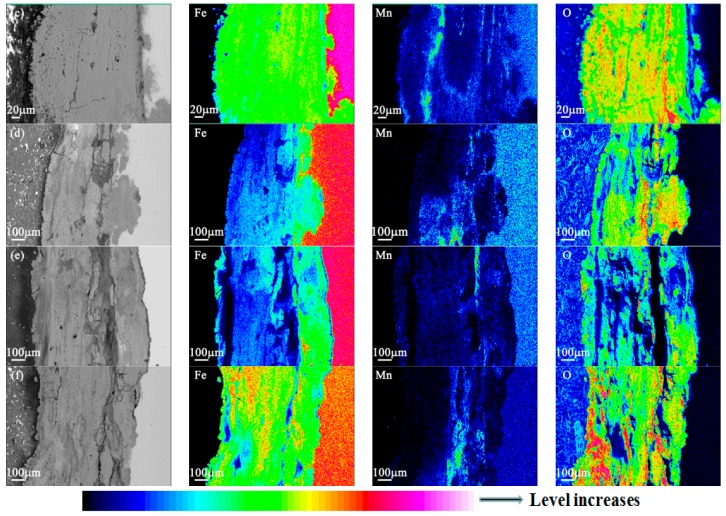
The main elemental distribution of the corrosion products formed in the tested medium-Mn steel after different immersion durations: (**a**) 24 h; (**b**) 72 h; (**c**) 168 h; (**d**) 288 h; (**e**) 432 h; and (**f**) 600 h.

The EPMA images of the tested medium-Mn steel corresponding to the different corrosion times presented in Figure 5 shows the main elemental distribution of the rust formed after cyclic wet/dry accelerated corrosion testing with a 3.5 wt % NaCl solution. After 24 h corrosion (Figure 5a), Fe and Mn elements were oxidized and concentrated in the outer rust film. The outer rust layer contained large amounts of Fe oxides and some Mn-rich compounds. The corrosion products between the outer rust film and the matrix presented a diffuse distribution, it indicated that the rust was forming with the corrosion solution and oxygen and this moment was an accelerated corrosion stage from this time point. After 72 h corrosion, a completed oxidation rust layer is presented in Figure 5b, and the Mn was enriched in the rust layer from the matrix to the outer rust layer. At this time point, the rust layer was completely dominated with the Fe corrosion products and the Mn-rich compounds and the corrosion rate reached the peak. After 168 h corrosion, the density of the Fe element increased again and the Mn migrated to the outer rust film (Figure 5c). After 288 h corrosion (see Figure 5d), the rust layer close to the matrix side was relatively dense and the outer rust layer was loose. Meanwhile, the Mn-rich compounds can be found in the outer rust layer. Similar to the initial corrosion, the corrosion process in the outer rust film was affected by the Fe and Mn. The corrosion rate decelerated due to the existence of the dense inner rust layer. After 432 h corrosion (Figure 5e), the elemental distribution in the rust layer was similar to that after 288 h corrosion. However, the Mn element was enriched near the midline of the rust layer. It was speculated that the existence of the anti-corrosion elements could cause this phenomenon due to the Mn-rich compound being fixed in the midline of the rust layer. Therefore, the rust layer achieved a stable cross-sectional morphology and elemental distribution after 600 h corrosion (Figure 5f).

**Figure 6:**
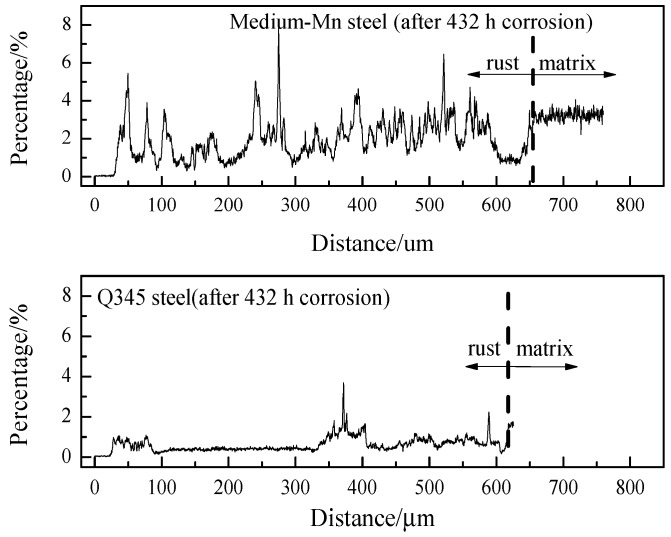
The typical EPMA line scanning results of Mn distribution in the rust layer of the tested medium-Mn steel and the Q345 steel after 432 h of corrosion.

Figure 6 shows the EPMA line scanning results of Mn distribution in the rust layer of the medium-Mn steel and the Q345 steel after 432 h of corrosion. The atomic characteristic absorption intensity from the EPMA results reveals that the Mn was enriched in the rust layer with a content of ~8% in the medium-Mn steel, which was higher than that of the rust layer in the Q345 steel. It was speculated that the instability of Mn concentration in the rust might produce certain adverse effects on the corrosion behavior in the testing of the medium-Mn steel.

### 3.5. X-ray Photoelectron Spectroscopy

**Figure 7:**
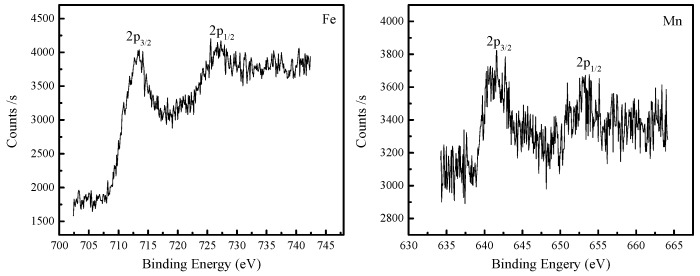
XPS spectra of Fe and Mn elements in the rust layer on medium-Mn steel after 432 h in a 3.5 wt % NaCl solution.

The chemical composition of the rust layer on medium-Mn steel was measured by an X-ray photoelectron spectroscopy system. However, Fe and Mn elements were obviously detected with XPS measurement compared with other alloy elements, as shown in Figure 7. The peaks of Fe 2p_3/2_ and Fe 2p_1/2_ were concentrated at 711.4 and 724.6 eV, which confirmed the existence of the oxidation state of trivalent Fe ions in the rust layer [14]. In addition, the peaks of Mn 2p_3/2_ and Mn 2p_1/2_ were observed at 642.3 and 654.0 eV, respectively. It identified the bivalent Mn ion oxidation state in the rust layer [15]. Hence, the results mentioned above further identify the Mn-rich compound composed of MnFe_2_O_4_ and Mn_3_O_4_ in the rust layer during the corrosion process.

### 3.6. Corrosion Process of the Tested Medium-Mn Steel

**Figure 8:**
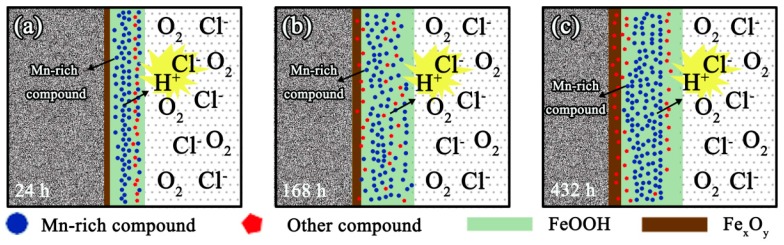
Conceptual model of the corrosion process of the tested medium-Mn steel: (**a**) 24 h; (**b**) 168 h; and (**c**) 432 h.

From the observed results and comparison with the Q345 steel on the corrosion process, it can be inferred that the addition of Mn can increase the corrosion rate with the corrosion process. The conceptual corrosion model of the tested medium-Mn steel is presented in Figure 8. In our experiment, the electrochemical reaction occurred in a near-neutral pH environment from a 3.5 wt % NaCl electrolyte solution. Under such conditions, Fe(II) complexes that form during the initial anodic dissolution were oxidized to FeOOH, the formation of which was in favor of chloride-saturated environments [16]. In addition, Fe_x_O_y_ acted as another main corrosion product during the entire process. These compounds can be formed due to the transformation of the FeOOH phase; this reduction reaction was favorable because the oxygen reduction process could not take place as a result of the limited supply of oxygen at the rust–metal interface [17,18]. The Fe_x_O_y_ constituted the final dense rust near the inner layer under the simulated seawater environment. Equilibrium equations for the stable range of Fe_3_O_4_ during the corrosion process were listed in Equations (2)–(4) [19,20]. Furthermore, the FeOOH might appear in two states during the corrosion process: lepidocrocite (γ-FeOOH) and goethite (α-FeOOH). These might originate from the Fe_x_O_y_ near the outer rust layer, and the transformation of Fe_x_O_y_ to FeOOH might take place in the porous or cracked locations.

3Fe^2+^ + 4H_2_O = Fe_3_O_4_ + 8H^+^ + 2e (2)

3FeO + H_2_O = Fe_3_O_4_ + 2H^+^ + 2e (3)

2Fe_3_O_4_ + H_2_O = 3Fe_2_O_3_ + 2H^+^ + 2e (4)

Previous investigations [21] have shown that a relatively high Mn content will result in a high corrosion current density in the potentiodynamic polarization test. Our results showed that the high content of Mn element can increase the corrosion rate by promoting the anodic sensitivity, which was similar to one reported by Eliyan et al.’s [22] study on the effect of the chloride content on corrosion of high strength steel. In our study, the effect of Mn content would be better to promote the hydrogen evolution during the corrosion process which was normally accompanied by charge transfer [22,23]. Furthermore, a better understanding was revealed in our experiments on the effects of Mn element in the rust layer as well as the interaction during the corrosion process.

Through a comparison between the alloy elements in the tested medium-Mn steel and the Q345 steel, it can be found that the tested materials contained some amount of anti-corrosion elements (Ni, Mo, Cr). However, the main difference of the two tested steels was the content of Mn. Mn might produce Mn-rich compounds (oxides and hydroxides) during the whole corrosion process. Some specific reactions were introduced into the electrochemical reaction, as shown in Equations (5)–(8) [19,20,24,25].

Mn^2+^ + 2FeOOH = MnFe_2_O_4_ + 2H^+^ (5)

2Fe_3_O_4_ + 3Mn^2+^ + 4H_2_O = 3MnFe_2_O_4_ + 8H^+^+ 2e (6)

3Mn(OH)_3_^−^ + 2Fe_3_O_4_ + H^+^ = 3MnFe_2_O_4_ + 5H_2_O + 2e (7)

3MnFe_2_O_4_ + 4H_2_O = 6FeOOH + Mn_3_O_4_ + 2H^+^ + 2e (8)

The generation of the Mn-rich compounds can produce adverse effects during the corrosion process. In terms of the above corrosion process, the effects of Mn-rich compounds were mainly manifested in two aspects: (1) Mn-rich compounds (such as MnFe_2_O_4_ and Mn_3_O_4_) can usually be used as an anode material as they have a spinel structure that was helpful for H^+^ to intercalate/deintercalate and their formation processes were accompanied by a higher H^+^ generation (from Equations (5)–(8)) [21,26,27]; and (2) the free energy of Mn-rich compounds were higher than other corrosion products during the corrosion process (Table 2); thus, it can exhibit higher spontaneous electrochemical reactivity.

The anti-corrosion alloying elements (Cu, Mo, Ni, and Cr) in medium-Mn steel can enhance corrosion resistance and weaken the adverse effects of elemental Mn. A lower content of Cu in the designed steel may improve corrosion resistance by forming CuO_x_ compounds that provide nucleation sites during the early stage of the corrosion process. An increase in the nucleation rate can inhibit the corrosion rate [28]. The addition of Mo decreased the corrosion rate by selective cation permeability via the formation of the MoO_4_^2−^ ion [6,7]. Ni promoted corrosion resistance in steel in amounts less than 0.5% [28,29]. It improved corrosion resistance by stabilizing the structure of the protective Fe_x_O_y_ phase [28,30]. Cr nucleates Cr compounds (oxides and hydroxides) that can act as a barrier to prevent the infiltration of the corrosive solutions and oxygen during the corrosion process [31,32]. The synergy effects of the above alloy elements can regulate the corrosion process of the tested medium-Mn steel and lead to its corrosion rate being close to that of the Q345 steel.

## 4. Conclusions

The following conclusions can be drawn from this work:

(1) Through a comparison of medium-manganese steel and Q345 steel on their corrosion behavior in a 3.5 wt % NaCl solution, the medium-Mn steel exhibited lower corrosion resistance due to the adverse effect of Mn. Mn-rich compounds presented adverse effects during the corrosion process.
(2) The two tested steels had similar corrosion behavior. Moreover, the rust morphologies of the Q345 steel were much denser than that of the medium-Mn steel. The corrosion rate was regulated because of the existence of the anti-corrosion elements in the medium manganese steel.

## Figures and Tables

**Table 1:** Chemical compositions of the specimens (in mass %).

Samples	C	Si	Mn	P	S	Al	Mo	Ni	Cr	Fe
Medium-Mn Steel	0.04	0.2	5.5	0.005	0.003	0.03	0.22	0.30	0.39	Bal.
Q345 Steel	0.17	0.32	1.38	0.019	0.009	0.04	-	-	-	Bal.

**Table 2:** Thermodynamic data of used species during the corrosion process of the tested medium-Mn steel.

Species	Δ*G*° (298.15 K) (J·mol^−1^)	Source	Species	Δ*G*° (298.15 K) (J·mol^−1^)	Source
H_2_	0	[25]	O_2_	0	[25]
H^+^	0	[25]	H_2_O	−237,191	[20]
Mn^2+^	−228,100	[20]	Mn(OH)_3_^−^	−744,200	[25]
MnFe_2_O_4_	−1,121,790	[25]	Mn_3_O_4_	−1,283,200	[25]
Fe	0	[25]	Fe^2+^	−78,900	[25]
FeO	−256,354	[25]	Fe_2_O_3_	−740,986	[25]
FeOOH	−485,300	[19]	Fe_3_O_4_	−1,015,450	[25]
